# Computer Simulation of Cellular Patterning Within the *Drosophila* Pupal Eye

**DOI:** 10.1371/journal.pcbi.1000841

**Published:** 2010-07-01

**Authors:** David E. Larson, Ruth I. Johnson, Maciej Swat, Julia B. Cordero, James A. Glazier, Ross L. Cagan

**Affiliations:** 1 The Genome Center at Washington University, St. Louis, Missouri, United States of America; 2 Department of Developmental and Regenerative Biology, Mount Sinai Medical School, New York, New York, United States of America; 3 Biocomplexity Institute and Department of Physics, Indiana University, Bloomington, Indiana, United States of America; 4 The Beatson Institute for Cancer Research, Colorectal Cancer and Wnt Signaling Group, Glasgow, United Kingdom; University of California, San Diego, United States of America

## Abstract

We present a computer simulation and associated experimental validation of assembly of glial-like support cells into the interweaving hexagonal lattice that spans the *Drosophila* pupal eye. This process of cell movements organizes the ommatidial array into a functional pattern. Unlike earlier simulations that focused on the arrangements of cells within individual ommatidia, here we examine the local movements that lead to large-scale organization of the emerging eye field. Simulations based on our experimental observations of cell adhesion, cell death, and cell movement successfully patterned a tracing of an emerging wild-type pupal eye. Surprisingly, altering cell adhesion had only a mild effect on patterning, contradicting our previous hypothesis that the patterning was primarily the result of preferential adhesion between IRM-class surface proteins. Instead, our simulations highlighted the importance of programmed cell death (PCD) as well as a previously unappreciated variable: the expansion of cells' apical surface areas, which promoted rearrangement of neighboring cells. We tested this prediction experimentally by preventing expansion in the apical area of individual cells: patterning was disrupted in a manner predicted by our simulations. Our work demonstrates the value of combining computer simulation with *in vivo* experiments to uncover novel mechanisms that are perpetuated throughout the eye field. It also demonstrates the utility of the Glazier–Graner–Hogeweg model (*GGH*) for modeling the links between local cellular interactions and emergent properties of developing epithelia as well as predicting unanticipated results *in vivo*.

## Author Summary

During development, organs are assembled through a complex combination of cell proliferation, programmed cell death, cell movements, etc. These aspects of tissue maturation must be achieved with a limited gene set—to achieve complexity, tissues utilize patterning mechanisms. That is, “rules” are used to create heterogeneity in initially homogeneous cell populations. A large number of genes and cell biology mechanisms have been uncovered that mediate this process but we have a limited understanding of how these factors act together to generate the large-scale patterns necessary to create a useful organ. Here, we combine computational modeling with *in situ* experiments in the developing *Drosophila* eye to explore these issues. Computer modeling is often criticized for describing known outcomes. We demonstrate how the Glazier–Graner–Hogeweg model can successfully predict surprising outcomes contradictory to models that emerged from our previous studies. We then validated these predictions in the developing eye. These mechanisms, which include the importance of dynamic nuclear movements, may prove generally important in directing cells into their proper niches as developing epithelia mature.

## Introduction

Epithelial patterning, in which cells assume required positions within emerging epithelia, is essential to the development of all animals. Such patterning results from local interactions that correctly localize each cell using limited molecular information. Simple patterns can employ a single surface factor, often adhesion molecules such as cadherins [1], [2]. Mathematical models and computer simulations of these processes based on local reduction of free-energy can replicate experimentally observed cell shapes within epithelia as diverse as embryonic germ layers and *Drosophila* ommatidial patterns [3], [4], [5], [6], [7], [8]. However, these models do not address cell placement, which commonly plays a key role in producing functional tissues for example in the mammalian and insect retinas [9], [10]. Recently, we proposed that multiple adhesion molecules expressed in precise spatial patterns can generate more complex patterns via local energy minimization [11]. Such models self-organize based on a small number of cell and global properties. However, we did not verify that such forces could control the arrangement of cells in a complex pattern [11].

**Figure 1:**
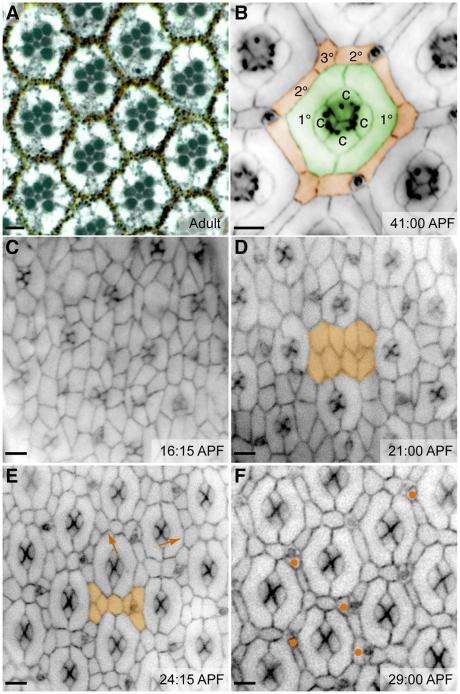
Patterning the fly eye. (**A**) Cross-section of an adult eye reveals the precise hexagonal arrangement of ommatidia. The reddish pigment granules are contained within the interweaving 2°/3° hexagonal lattice. Membranes highlighted with methylene blue. (**B–F**) Images illustrating progressive stages of pupal eye development; times are as indicated. (**B**) The completed pattern. Cell types are false colored for clarity. An ommatidial core (*OC*) of cone cells (*c*) and primary pigment cells (*1°*) is highlighted in green. Orange highlights the hexagonal lattice of secondary (*2°*) and tertiary (*3°*) pigment cells; bristle groups alternate with 3°s at the vertices. (**C**) At the beginning of pupal patterning, cone cell clusters are arrayed within a large collection of undifferentiated cells. (**D**) 1°s envelop the cones and isolate them from the rest of the developing eye. Multiple layers of IPCs remain between developing ommatidia (orange highlights a sample region). (**E**) Cell rearrangement generates a single layer of cells between ommatidia (orange). At this stage, the cell number is noticeably reduced and 3°s begin to appear (arrows). (**F**) Further death and rearrangement generate a single cell (2°) on each side of the hexagon and a single cell (3°) at each vertex. At this stage a few extra cells remain (•). Our simulations model patterning that occurs between the stages shown in (**E**) and (**F**) and further refined to (**B**). Bars represent 5 µm.

The *Drosophila* eye is a striking example of cell placement, in which every cell has a stereotyped position. It is a mosaic of approximately 750 precisely organized *ommatidia* (Figure 1A). Each ommatidial core (*OC*) contains eight photoreceptor neurons and six glial-like support cells (two *primary pigment cells* (*1°*s) and four *cone cells* (*c*); Figure 1B) that aggregate in the larval and early pupal eye anlage. To explore assembly of the cone cell quartet Kafer *et al.* used Glazier-Graner-Hogeweg (*GGH*)-based simulations to model experimental evidence that, within each OC, cone cells assemble into precise quartets through homophilic Cadherin-based adhesion and tension in the cells' actin cortices [3]. Cone cell assembly, similar to the packing of soap bubbles, is therefore an example of short-range, surface-energy-driven aggregation of like cells into a cluster. This local phenomenon demonstrates the ability to utilize local cell-cell angles as an indication of the local processes that assemble discrete cell clusters [3], [6]. Considering local cell shape changes leaves open the broader question, however, of how multiple processes— including the dynamic aspects of cell adhesion, cell death, cell movement, and cell shape changes— act in concert to achieve progressive, coordinated patterning across an epithelium. Here we focus on the emergence of a complex, interweaving array that organizes the visual field.

After OC assembly, an interweaving hexagonal lattice of *secondary* (*2°*) and *tertiary* (*3°*) *pigment cells* (collectively termed inter-ommatidial pigment cells, *IPC*s) and sensory bristles (Figure 1F) develops that re-organizes the retinal field. Cell re-arrangements begin at the tissue's surface [12] and the lattice eventually extends the length of the eye field to optically isolate neighboring OCs. The emergence of this IPC lattice during pupal development requires regulated cell adhesion, directed cell movement, PCD, and changes in cell shapes (reviewed in [13], [14], [15], [16]). IPC patterning provides a useful model for exploring the patterning mechanisms required to assemble emerging epithelial tissues. This paper simulates the known parameters that organize cells into a locally repetitive pattern that connects across an entire tissue, an aspect of development that has not been previously modeled.

Ordered assembly of the IPC lattice requires members of the Nephrin superfamily of proteins, which include the heterophilic type I transmembrane proteins Hibris (*Hbs*) and Roughest (*Rst*; [11], [17]). Experimentally, altering the levels of Hbs and Rst in the developing pupal eye disrupts patterning of the IPC lattice [11]. By analogy with Steinberg's *Differential Adhesion Model* of homophilic adhesion [1], we proposed a *Preferential Adhesion Model* of the assembly of the hexagonal lattice based on heterophilic adhesion between cells expressing Hibris (1°s, cone cells) and Roughest (neighboring IPCs; [11]). In this model, IPCs reduce their contacts with each other and enhance their contacts to neighboring 1°s to promote Hbs/Rst binding, reducing their local free energy.

We tested this model using simulations based on the Glazier-Graner-Hogeweg model (*GGH*, also known as the Cellular Potts Model, *CPM*), a multi-cell methodology that allows for non-uniform cell shapes. The GGH successfully reproduces much of the phenomenology of differential adhesion-based cell sorting [5], [18], [19]. We show that simulations using this methodology can replicate the step-wise patterning of ommatidia within the pupal eye field. Surprisingly, our simulations suggest that preferential adhesion is not sufficient to position IPCs. As anticipated, the simulations demonstrated that selective cell death provides robustness to patterning, in particular the movement of 3°s into their final niche. More surprising, robust simulated patterning required a previously unappreciated mechanism: steady expansion of the OCs' surface profiles that effectively ‘crowd’ neighboring IPCs into a proper hexagonal array. Following this suggestion, we used over-expression of the ubiquitin ligase Smurf to prevent normal surface expansion of the OCs; neighboring IPCs failed to pattern properly as our simulations predicted. Thus, our *in silico* predictions suggested the importance of specific mechanisms in development and led to new experiments that confirmed the importance of these mechanisms, demonstrating that developmental simulations can predict novel mechanisms and indicate specific experiments required to assess them *in vivo*.

## Results

### Patterning of the pupal eye

The *Drosophila* pupal eye is a post-mitotic, pseudo-stratified epithelium within which every cell eventually achieves a stereotyped position. OCs assemble fully by the young pupal stage: photoreceptor neurons and cone cells emerge in the larva, and the 1°s complete each core by enwrapping the cone cells at the surface by 21–22 hours after puparium formation (*APF*; Figure 1D). Ablation studies have demonstrated that the photoreceptors do not contribute to the surface hexagonal patterning [20]. Between 21 and 24 hours APF, cell rearrangements and programmed cell death refine the remaining interommatidial precursor cells (*IPC*s) to a single row (Figure 1E; [12], [16], [21]). By 30 hours APF, further cell rearrangements and deaths pare the pattern down to a single cell for each side of the hexagon and a single cell at each vertex (Figure 1F).

Roughly one-third [22] of the cells present at the beginning of pupal development die via PCD before the eye fully patterns. Cell division is essentially complete by this stage, so the hexagonal pattern primarily results from cell rearrangements and deaths.

### Developing a GGH-based simulation

Our simple two-dimensional simulation of eye development implements the free-energy mechanisms proposed previously [11] using CompuCell3D [23], [24], an open-source implementation of the GGH. We therefore focused on patterning at the apical surface of the epithelium for two reasons. First, as the pupal eye develops, cell-cell contacts begin at apical surfaces then extend basally through the epithelium [12]. Second, most factors known to initiate patterning are localized to the apical surface including Notch, EGFR, Hibris, Roughest, Cindr, Pyd, *etc.* [11], [25], [26], [27], [28].

The GGH is a multi-cell model that has accurately reproduced cell sorting based on differential cell adhesion [5], [18], [19]. It represents each ‘cell’ as a collection of points in a two-dimensional grid ([29], [30]; see Methods). The spatial configuration and physical properties of these cells determine the ‘energy’ landscape of the pattern. Cells move by extending and retracting apical extensions, favoring changes that reduce the local pattern energy. The rate of these extensions determines the timescale of the simulation, measured in *Monte Carlo Steps* (*MCS*). In our simulations we used MCS to represent developmental time; we define the relationship between the two below.

### Mechanism 1: multiple (two) cell types

Laser ablation studies demonstrated that the key IPC patterning interaction is between (i) the cone cells and 1°s of the OCs and (ii) IPCs [20]. In defining IPCs we noted that previous experiments demonstrated (i) bristles are nonessential for patterning and (ii) 2°s and 3°s are molecularly indistinguishable, differing only in their positions within the hexagonal pattern (Figure 1B; [20] [31], [32]). We therefore simulated two cell types: OCs and IPCs. We assessed patterning accuracy by monitoring the emergence of 2°s and, separately, 3°s.

### Mechanism 2: type-dependent cell-cell adhesion

In the GGH, adhesion takes the form of a boundary energy (*J*; see Methods). A higher boundary energy corresponds to weaker cell-cell adhesion. While absolute values for experimental cell-cell adhesion strengths are unknown, previous experiments have found stronger adhesion at OC:IPC contacts than at IPC:IPC contacts [11]. Stronger OC:IPC adhesion prevents contact between neighboring OCs. A ring of IPCs bounds the eye field. Based on these observations, we initially assumed a hierarchy of contact energies: *J*_OC, OC_>>*J*_OC, Medium_>>*J*_IPC, Medium_>*J*_IPC, IPC_≥*J*_IPC, OC_, where the ‘Medium’ cell type represented the intracellular space surrounding the edge of the developing imaginal disc, allowing tissue expansion. We initially assumed OC:IPC adhesion to be stronger than IPC:IPC adhesion to prevent OC fusions; we later tested this assumption as well. Finally, a broad array of values for OC:OC adhesion were tested and yielded similar results.

### Mechanism 3: intrinsic cell motility

Pupal epithelial cells have an intrinsic cytoskeleton-driven motility that appears in continuous live imaging as jostling— short range undirected movements— within the pupal eye field [33]. This motility likely depends on interactions between surface junction factors and the actin cytoskeleton [34], [35]. The GGH represents this intrinsic motility by the parameter *T* (see Methods). A larger *T* corresponds to higher cell motility in the simulation [36].

### Mechanism 4: programmed cell death (PCD)

Though the mechanisms that direct PCD of specific IPCs are not fully understood, PCD depends on levels of Rst [14], [32], [37], [38]. In the GGH, we simulate PCD by changing a cell's target area (*A*_T_) to 0 [39], causing the cell to shrink and disappear.

We implemented a simple PCD rule based on two experimental observations: (i) 1°s are necessary for cell survival (ablation of 1°s leads to PCD of all adjacent IPCs [20]) and (ii) reduction of a cell's apical surface area predicts PCD [32]. Regarding the latter, reduction of the apical profile of emerging IPCs was sufficient to increase the likelihood of an emerging IPC's death [11]. In our simulations, the probability that an IPC ‘died’ via PCD increased when its contact with OCs became smaller than a threshold length (*L*; see Methods), thus mimicking the observed biological behavior. Experimentally, IPCs in the periphery of the pupal eye do not undergo PCD in the young pupa [22], [40]; we therefore prevented the IPCs at the edges of simulated eyes from dying regardless of the extent of their contact with the OCs.

### Mechanism 5: apical surface expansion of OC profiles

**Figure 2:**
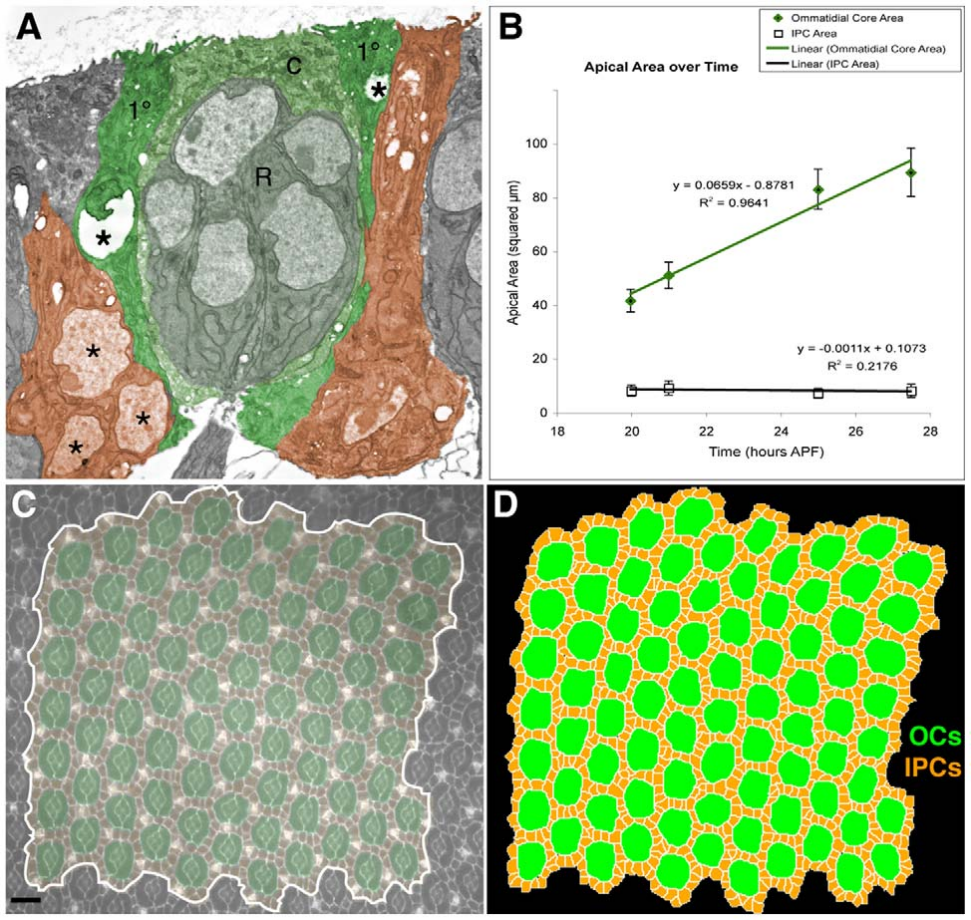
Apical OC expansion and initial simulation inputs. (**A**) TEM of an ommatidium in an 18 hour APF eye. The central photoreceptors (R), cone (c) and 1° cells are pseudo-colored green. Note that the left 1° nucleus (asterisks) is rising, that on the right (only partially seen) is already higher resulting in a larger apical profile; the neighboring 2° nuclei are basal, these cells have larger basal footprints. Select 2° cells are pseudo-colored orange. (**B**) The OC increases in apical surface area (µm^2^) over time as measured from live images. The error bars indicate one standard deviation. (**C, D**) Example of a 22 hour APF pupal eye stained with an E-cadherin-specific antibody to visualize surface cell boundaries. This image was traced to provide a starting point for the simulations, which were then run multiple times as noted. The region used in the tracing is false colored as in Figure 1. Bars represent 10 µm.

As the developing pupal eye matures, the apical cell-surface profile (that is, the cross-sectional area) of the OCs increases roughly linearly in time due to apical migration of cone and 1° cell nuclei (Figure 2A, compare Figure 1D, E and F; [41]). In our experiments, the average area of each OC approximately doubled between 22:00 and 27:30 hours APF, while the apical area of the IPCs remained approximately unchanged (Figure 2B). To simulate this steady increase in apical IPC profiles we observed *in situ* we doubled the target area and target perimeter of simulated OCs in a linear progression (Table S1).

### Simulation of wild-type development

We traced micrographs of wild-type eyes at 23:00–24:30 hours APF (Figure 2C, 2D; see Methods). By this stage 1°s have fully enveloped each ommatidium, the pattern of 2°s has advanced but is not complete, and most 3°s have yet to move to the vertices. We used a representative tracing as the initial condition of our simulations, then ran for 50,000 MCS. We initially tested several values for adhesion energies and temperature to establish the range that yielded patterns closest to experiment (Table S2). The number of (i) IPCs that established themselves correctly in vertices as 3°s and (ii) single cells that occupied a 2° cell locus provided straightforward measures of pattern precision. Importantly, all wild-type patterning was essentially complete by 20,000–30,000 MCS. Thus 10,000 MCS is equivalent to approximately 5.5 hours. We ran simulations to 50,000 MCS to ensure that we were observing a pattern in equilibrium and to provide additional steps for simulations employing ‘mutant’ parameters.

To determine values for adhesion, we assumed the hierarchy described above and tested various values (Table S2) to yield cell shapes and placement similar to those observed in wild-type retinae (Table S3). Altering the cell mobility parameter over a broad range did not affect the positions of cells at the end of our simulation: 3°s formed between 25<*T*<150, so we selected an intermediate value of *T* = 60 for further simulations. By varying the threshold contact length for induction of PCD over a range of motilities, we found that *L* = 16 generated ommatidia with the proper number of cells with occasional surplus IPCs that were confined to 2° niches, an observation consistent with the occasional ectopic 2°s observed in mature wild type pupal eyes (Figure 1F). We delayed PCD until the simulation had run for 10,000 MCS to equilibrate cell sizes and remove any bias in cell death due to the initial configuration. However, delaying the onset of PCD did not affect the outcome of the simulations (data not shown).

**Figure 3:**
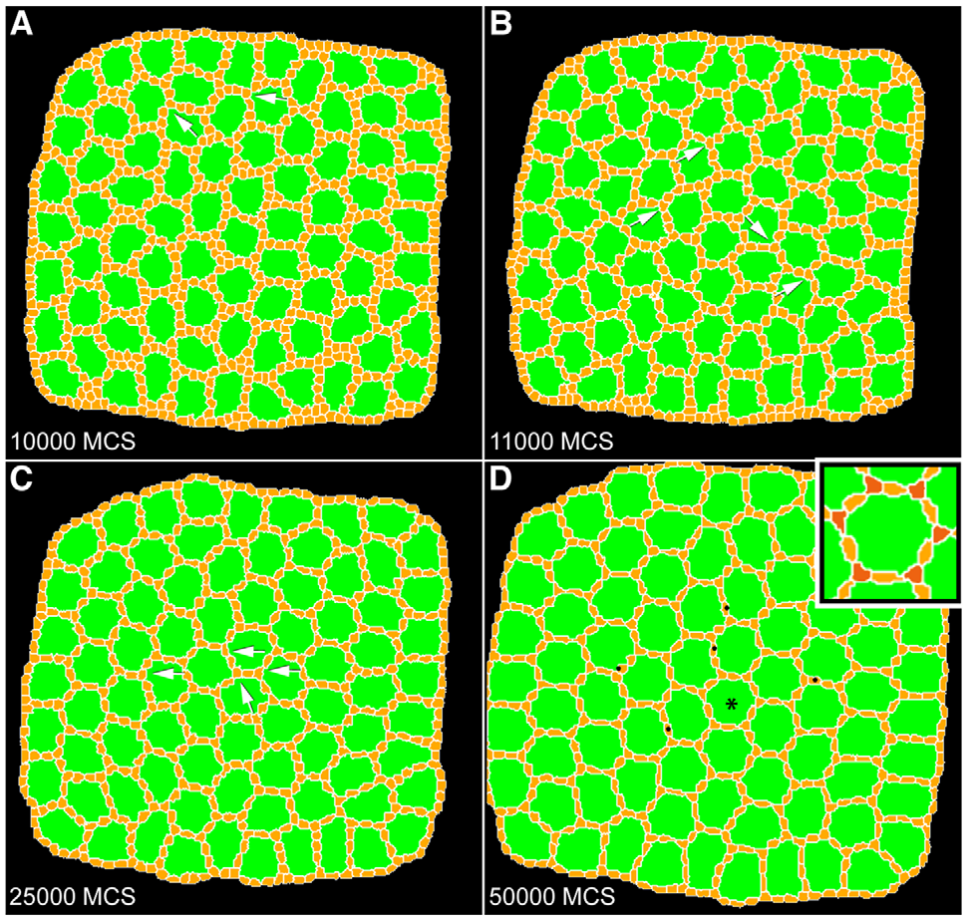
Simulation of wild-type development. (**A**) Image captured at 10,000 MCS exhibited numerous ectopic cells and few 3°s (arrows). Cell death has not yet been initiated. (**B**) Image from 11,000 MCS. Cell death has been initiated and there are fewer cells and more 3°s (arrows). (**C**) Image from 25,000 MCS. There are fewer cells and most central ommatidia have cells in the 3° locus (arrows). (**D**) Image from 50,000 MCS. There are a few extra 2°-like cells (•) and all central ommatidia show a complete complement of 3°s. Asterisk labels the ommatidium shown in inset with 3°s colored red.

Using an adhesion hierarchy in which IPCs adhered more to OCs (*J*_OC,IPC_ = 35) than to each other (*J*_IPC,IPC_ = 55; Table S3) produced a striking phenocopy of a wild type eye with a single 3° in most vertices (Figure 3 and Supplemental Video S1). This final pattern was consistent over multiple runs and reached a steady-state equilibrium after 50,000 MCS (equivalent to approximately 41 hours APF in the developing pupa). Most patterning was completed by 30,000 MCS, which corresponds to approximately 30 hours APF, mirroring the time frame of development *in vivo*.

### Simulation of mutations that alter cell death

**Figure 4:**
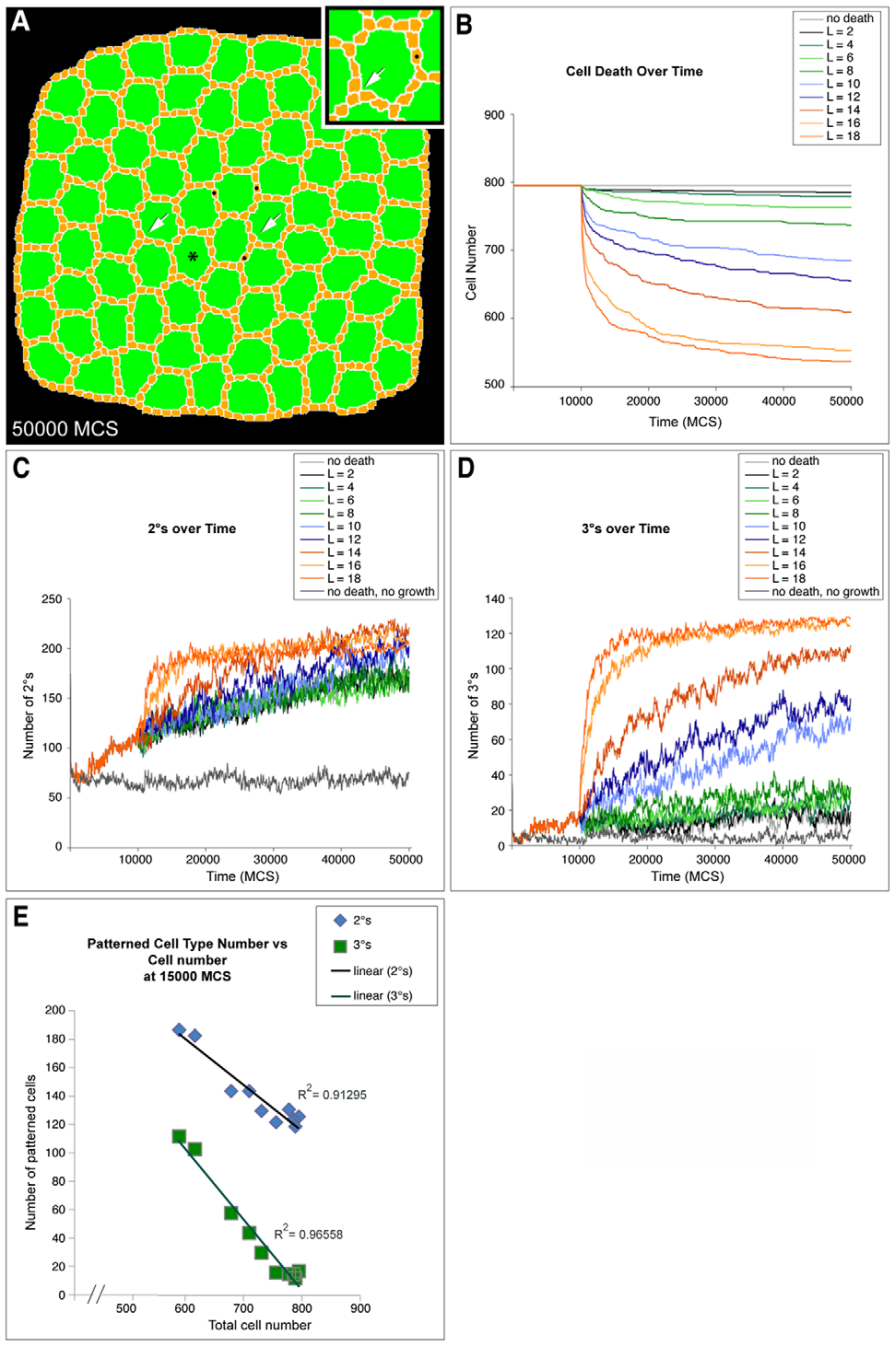
Secondary and tertiary formation depends on cell death. (**A**) Image captured at 50,000 MCS from a simulation of reduced cell death (L = 12) showed numerous ectopic, end-to-end 2°-like cells (•) and vertices with three cells in the 3° locus (arrows). Asterisk labels an ommatidium enlarged in inset. (**B**) Graph showing the decrease in cell number over time for different levels of cell death. L controlled the ‘strength’ of cell death as described in Results. Cell death was initiated at 10,000 MCS and each line represents the result of a single representative simulation from at least two repetitions. (**C**) Graph showing formation of 2°s over time for different levels of cell death. Each line represents the result of a single representative simulation from at least two repetitions. (**D**) Graph showing formation of 3°s over time for different levels of cell death. Each line represents the result of a single representative simulation from at least two repetitions. (**E**) Graph showing number of both 2° and 3°s at 15,000 MCS as cell death parameters are changed to increase the total cell number. Each point represents a different cell death parameter in the simulations from (B), (C) and (D).

Having determined parameters that replicated wild-type development, we next tested the contributions to patterning from different mechanisms by varying parameters from the wild-type values. To reduce PCD, we decreased the threshold length of OC:IPC contact required for survival, resulting in ectopic 2°-like cells consistently positioned end-to-end (Figure 4A, 4C). Further, we observed a direct correlation between the number of cells present in the simulation and (i) the number of single cells occupying a 2° cell locus (Figure 4C) and (ii) the number of 3°s eventually established (Figure 4D). As cell death was increased toward normal levels in the simulation the number of successfully established 2°s and 3°s increased as well (Figure 4E).

**Figure 5 pcbi-1000841-g005:**
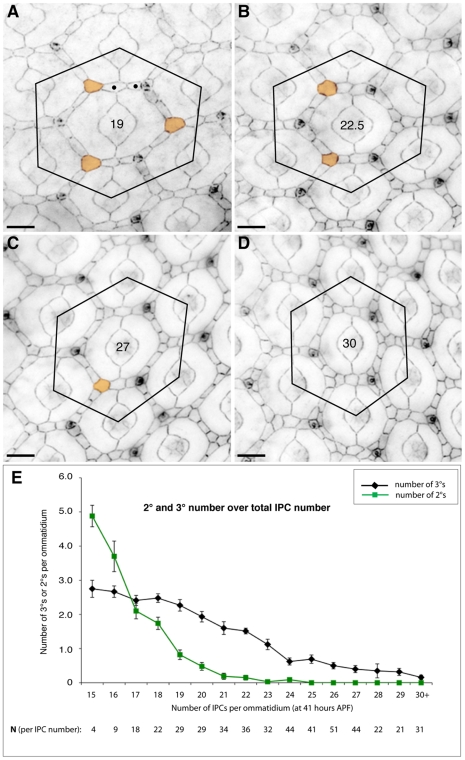
Inhibiting cell death impeded 2° and 3° formation *in vivo*. (**A–D**) Examples of ommatidia from *GMR-Gal4/UAS-Diap1* retinae. For cell counts all IPCs lying within the hexagonal outline as drawn were counted; cells partly within the hexagon were counted as a half-cell. 3°s were defined as contacting three 1°s (colored orange); a correctly specified 2° locus was defined as one occupied by only one cell; examples of end-to-end 2°-like cells are labeled with •. Total cell count for each ommatidium is indicated at its center. Our analysis included only ommatidia surrounded by three correctly positioned bristle groups. Bars represent 10 µm. (**E**) Graph showing the decrease in 2° (plotted in green) and 3° (black) cell number as IPC number increased. The total number of ommatidia analyzed (N) per data point plotted is indicated below the y-axis.

To test these predictions experimentally, we blocked PCD during pupal eye patterning with the caspase inhibitor DIAP1. Despite the greater number of IPCs, interommatidial cells still assembled into an interweaving hexagonal lattice as previously shown [37], [42]. Also as observed in our simulations, ectopic 2°s were found primarily end-to-end in experimental tissue (Figure 5A–D). PCD inhibition that produced three excess IPCs within a standardized region (see Methods, Figure 5) led to occasional misplacement of a single 2° cell and 3° vertex in pupal eyes. However when the number of IPCs exceeded 22 within this standardized region—an excess of 10 or more IPCs—we rarely (0.6%) observed the full complement of three correct 3°s and never observed six correct 2°s around an ommatidium. The number of 2°s and 3°s *in vivo* was progressively reduced as the number of IPCs increased: for example, nearly five correctly positioned 2°/3°s were observed in tissue with 15 IPCs per ommatidial region; this number decreased to less than one 2°/3° in regions with more than 24 IPCs (Figure 5E). The ability of our model to correctly predict this relationship provides further validation of our simulation parameters.

### Simulation of preferential adhesion

**Figure 6 pcbi-1000841-g006:**
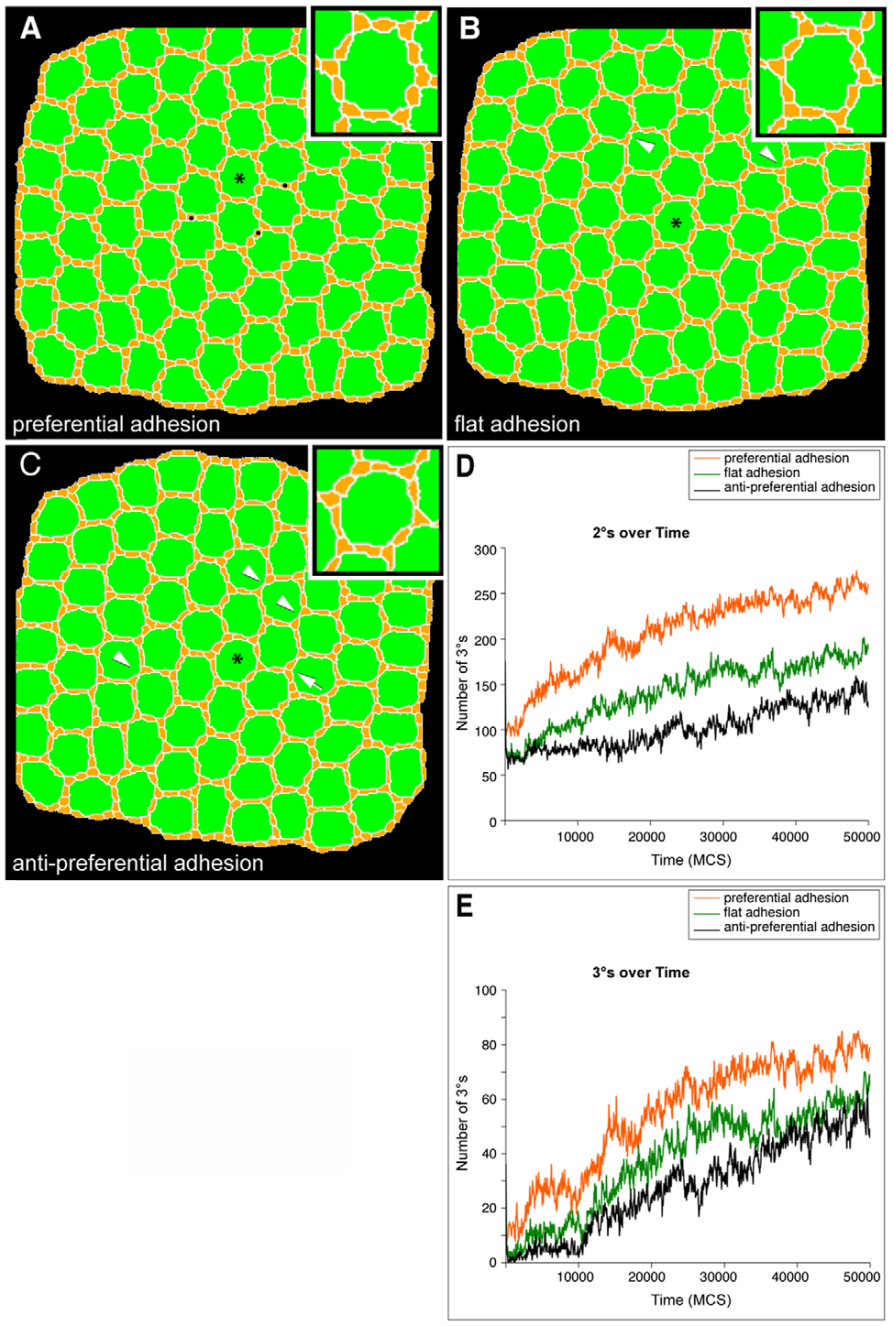
Adhesion did not affect formation of 2°s or 3°s in the presence of cell death. All images are final images from 50,000 MCS trials. (**A**) In a simulation in which IPCs were more adherent to OCs than to each other, 3°s and most 2°s emerged correctly at all central ommatidia. Several ectopic end-to-end 2°-like cells are indicated (•) (**B**) In a simulation in which OC:IPC and IPC:IPC adhesion was set as identical, 3°s still correctly emerged within all central ommatidia. An increase in cell death resulted in some missing 2°s (arrowheads). (**C**) 3°s also emerged within a simulation in which IPCs were more adherent to each other than to OCs; occasional loss of 3°s was observed (arrow). Increased cell death (PCD) resulted in missing 2°s (arrowheads). Asterisks label enlarged ommatidia (insets). (**D** and **E**) Graphs quantifying how in the presence of reduced cell death (L = 10), preferential adhesion most efficiently enhanced the ability of 2°s (**D**) and 3°s (**E**) to form. Each line represents the result of a single representative simulation from at least two repetitions. When OC:IPC adhesion was the same (‘flat adhesion’) or less (‘anti-preferential adhesion’) than IPC:IPC adhesion 2°s and 3°s still formed though less efficiently.

Our recent work has suggested that the preferential adhesion of IPCs (expressing Rst) to 1°s (expressing Hbs) is a major driving force in IPC patterning [11]. We assessed this ‘Preferential Adhesion’ model by simulating altered adhesion. We simulated three types of adhesion between OCs and IPCs: ‘preferential’ (*J*_OC,IPC_<*J*_IPC,IPC_), ‘flat’ (*J*_OC,IPC_ = *J*_IPC,IPC_), or ‘anti-preferential’ (*J*_IPC,IPC_<*J*_OC,IPC_). Remarkably, all three adhesion hierarchies led to correct or nearly correct assembly of 2°s and 3°s, though with decreasing levels of robustness (Figure 6). The reduced adhesion between IPCs and OCs in the ‘flat’ and ‘anti-preferential’ adhesion simulations led to decreased average IPC:OC contact length, increased number of cells dying by PCD, and increased frequency of defects due to missing 2° cells in (Figure 6B and C).

To check whether cell death masked the patterning effects of differential adhesion, we repeated the different adhesion simulations with a reduced rate of PCD that produced an intermediate number of 3°s. Under these conditions, ‘preferential’ adhesion resulted in only slightly better patterning than either ‘flat’ or ‘anti-preferential’ (Supplemental Video S2), as assessed by the number of correctly located 2°s and 3°s (Figure 6D, 6E) and the accuracy of the overall hexagonal patterning.

Our surprising conclusion is that *in silico* ‘preferential adhesion’ contributes to patterning robustness but that it is not sufficient to create the hexagonal pattern. We were unable to recapitulate our different *in silico* adhesion conditions *in vivo*, perhaps because manipulating the levels of Rst and Hbs led to confounding non-adhesion-related effects potentially due to aberrant signaling [11], [27], [28].

### Simulation of cell expansion

**Figure 7:**
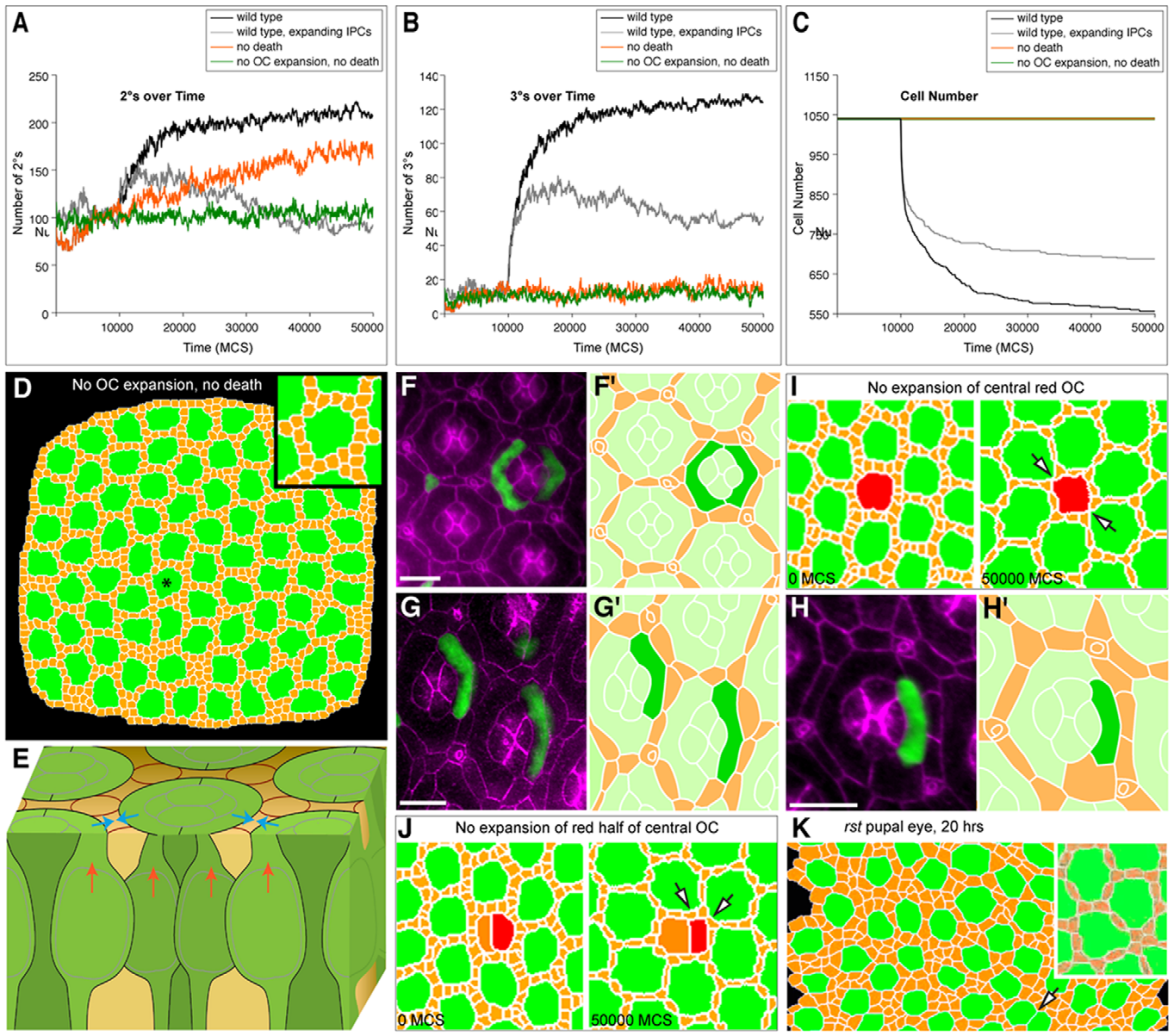
Progressive differences between IPCs and OC surface proportions promoted 2° and 3° formation. (**A** and **B**) Graphs showing formation of 2°s (A) and 3°s (B) over time for different size ratios between IPC and OC apical surface areas. Cell death was initiated at 10,000 MCS. The ‘wild-type’ curve indicates 2° and 3° formation when OC apical surfaces expanded but IPCs did not expand during progressive MCS. ‘Wild-type, growing IPCs’ indicates OCs expansion and IPC surface doubling. ‘No Death’ curve indicates expanding OCs with no cell death. ‘No OC Expansion’, No Death' curve indicates constant OC surface area (no expansion) and no cell death. (**C**) A graph indicating the decrease in cell number for the different simulations. Each line represents the result of a single representative simulation from at least two repetitions. (**D**) Image capture at 50,000 MCS from a simulation with no OC expansion nor cell death; the pattern fails to resolve; asterisk labels enlarged ommatidium (inset) (**E**) Three dimensional schematic emphasizing how the vertical movement of nuclei (red arrows) expands the 1°s' surface profiles (blue arrows), in turn laterally ‘crowding’ the neighboring IPCs into a hexagonal pattern. (**F, G** and **H**) Reducing OC expansion by expressing *smurf* introduced patterning errors *in vivo*. Bars represent 10 µm. (**F**) Ectopic expression of *smurf* in paired 1°s led to mild patterning defects, primarily in cell number. (**G** and **H**; corresponding tracings in **G′** and **H′**) Reducing growth of single 1°s (marked by GFP) reduced the apical surface profile (E-Cadherin shown in magenta); the neighboring IPC arrangement and 3° cell loci failed to properly resolve. (**I** and **J**) Central region of images captured at 0 MCS and 50,000 MCS in simulations in which (**I**) the entire central OC indicated in red or (**J**) only half of the OC was prevented from expanding. Arrowheads indicate defects also commonly observed *in vivo* – missing or ectopic cells. (**K**) Reduced *rst* activity (*rst^CT^*) led to consistently uneven IPC distribution in 20 hr APR eyes; within this tracing, an example of a rare fused ommatidium is indicated (arrow). *Inset*: our computer model consistently failed to pattern this *rst* tracing even after 50,000 MCS The failure to pattern using the same parameters as our wild type tracing indicates that the 20 hr APF *rst* eye field must already show differences with wild type tissue.

The failure of PCD plus preferential adhesion to fully account for patterning within our simulations suggested the importance of additional mechanisms. In re-assessing our experiments, we observed that ommatidial apical profiles expanded significantly as OC nuclei migrated apically throughout the patterning period, while IPC profiles remained roughly constant (Figures 1C–F, 2B). We therefore explored the contribution of OC surface expansion to 2° and 3° formation. Blocking OC expansion *in silico* led to indiscriminant death of IPCs (data not shown), since IPCs had very little available OC surface to bind to and thus died according to our PCD rules. As we discussed above, blocking cell death but retaining OC expansion permitted the emergence of at least some 2° and 3°s (Figure 7A, 7B). Eliminating both OC expansion and cell death, however, blocked emergence of nearly all 2° and 3° cells (Figure 4, 7A, 7B and 7D). This result indicates that expanding OCs play an obligate role in simulated IPC patterning.

If the expanding apical surface area of OCs relative to IPCs promotes 3° formation *in silico*, then increasing both OC and IPC sizes concurrently should inhibit lattice patterning. Having IPCs slowly double in size over the course of the simulation as OCs grew (Supplemental Video S3, Table S1) significantly reduced the number of correctly specified 2° and 3°s (Figure 7A, 7B). This reduction did not appear to result from the slight decrease in cell death we observed with expanding IPCs (compare Figure 4B to [Fig pcbi-1000841-g005] [Fig pcbi-1000841-g006] Figure 7C).

### Assessing the importance of cell expansion in the developing pupal eye

As schematized in Figure 7E, the size of a cell's apical surface profile is closely tied to how close its nucleus is to the surface: the nucleus makes up the large cross-sectional area of the cell (Figure 2A; [43], [44]). To test whether the changing relative sizes of OCs and IPCs contributes to eye patterning *in vivo*, we identified a mutant in which the surface profiles of 1°s failed to properly expand. The *smurf/lack* locus encodes an ubiquitin ligase with several functions including degradation of the cytoskeleton regulator Rho1 [43], [44]. Expressing ectopic *smurf* in isolated 1°s led to an autonomous reduction in their apical surface areas (Figure 7F, 7G, 7H). Attempts to manipulate cell size by modulating insulin signaling or nuclear positioning by perturbing *marbles* failed to consistently alter apical surface areas (data not shown).

As our simulations predicted, reducing the apical surface area of one or more 1°s led to local mis-patterning of the neighboring, genotypically normal IPC lattice. In 69% of ommatidia with isolated *smurf*-expressing 1°s, neighboring IPCs patterned incorrectly (*N* = 87). In control *GFP*-expressing 1°s, by comparison, IPC errors were observed in 3.5% of ommatidia (*N* = 502, not shown). Patterning defects included loss of IPCs, mis-patterned 2°s, and a reduction in proper 3°s (Figure 7G, 7H). We observed analogous defects in GGH simulations in which half of the OC was designed to not expand (Figure 7J, Supplemental Figure S1).

Interestingly, symmetric OC reduction led to milder IPC patterning defects in our simulations, suggesting that balanced expansion across the local field is necessary for proper patterning (Figure 7I). Again consistent with the GGH prediction, expression of *smurf* in both 1°s within an ommatidium had a less severe effect on patterning (Figure 7F). In addition to further validating GGH predictions, this result also indicates that the *in vivo* IPC defects were not due to unanticipated effects of reduced *smurf* in 1°s. Together, our data support the view that properly expanding OCs are a central component of IPC patterning.

### Roughest may affect earlier stages

While our simulations suggest that preferential adhesion contributes to the formation of 2° and 3° cells within the eye, we also observed that these cells can pattern without preferential adhesion. This result raises the question as to why *rst* mutant phenotypes are stronger than the mild effects generated in our ‘flat’ (*J*_OC,IPC_ = *J*_IPC,IPC_) adhesive paradigm. If the model's prediction is correct regarding adhesion, the *rst* locus may have activities in addition to adhesion as proposed for Neph1 (*e.g.*, [45], [46], [47]) and/or it may also be required for earlier stages of patterning. To test the latter prediction, we traced a 20 hr APF genotypically *rst^CT^* mutant eye and used it as the starting point for our standard wild type simulation. Interestingly and unlike wild type tracings, simulations using tracings of *rst^CT^* eyes as their initial conditions consistently yielded uniformly incorrect final patterns even after 50,000 MCS (Figure 7K). Thus, modeling of *rst* mutant eyes predicts that *rst* acts prior to 20 hour APF, during stages when adhesion-mediated IPC movement is not thought to occur. Indeed, visual inspection of 20 hr APF *rst^CT^* mutant eyes indicated consistently abnormal OC spacing (*e.g.*, Figure 7K; data not shown).

## Discussion

Generation of a hexagonal lattice of 2°s and 3°s requires interactions between two different cell types. Using a relatively small number of physical mechanisms demonstrated to regulate patterning *in vivo*, we produced a cellular configuration that replicates the precision of the *Drosophila* IPC lattice *in silico*. Most gene mutations alter multiple mechanisms simultaneously, such as adhesion plus cell death (*e.g.*, *rst*, *pyd*; [11], [27], [28]); our simulations allowed us to isolate and examine each mechanism separately to explore its role and importance in patterning. In addition to supporting the central importance of selective programmed cell death and its relationship to 1°:IPC contacts, our simulations highlighted the importance of a previously overlooked mechanism, challenging our current understanding of how cells organize within the emerging eye epithelium. It has also caused us to re-evaluate the role played by *rst* in IPC patterning. Table S4 compares our simulations with *in vivo* results.

Although the parameters utilized for proto-2°s and proto-3°s were identical our simulations correctly introduced a single cell into each 3° niche, suggesting that the 3° fate results from cell sorting within the eye and does not necessarily reflect a distinct differentiation state. The lack of experimental molecular markers that distinguish 2°s from 3°s is consistent with this simulation result. Further, while blocking PCD experimentally had little effect on the overall pattern of the lattice [14], [32], [37], [38], our simulations suggested that PCD is a major driving force behind establishment of 3°s. In our simulations, the number of 3° errors was directly proportional to the number of ectopic IPCs. We confirmed the accuracy of these predictions by reducing PCD experimentally during patterning of the pupal eye (Figure 5).

Our simulations also indicated the importance of a novel patterning component: the progressive increase in the OC:IPC apical surface profile ratio. A role for this process in eye patterning has been neither previously suggested nor explored. Our results suggest that expansion of the ommatidial array helps generate a hexagonal pattern by ‘crowding’ IPCs into elongate shapes that encourage proper cell death and correct 2° and 3° formation (Figure 7E). Our model predicts that asymmetric changes in 1°s will alter this pattern while symmetric changes will exhibit milder effects (Figure 7I, 7J). We have validated this prediction experimentally (Figure 7F, 7G, 7H).

Nuclear movements are commonly tightly coordinated during tissue maturation. For example, during emergence of the developing brain, nuclei move in a coordinated fashion (‘nucleokinesis’) that both indicates and is perhaps necessary for progressive cell specification, cell cycle, and stratification (*e.g.*, [48], [49], [50], [51], [52]). Work in cultured neurons suggests this process requires dynamic interactions between components of the cytoskeleton, the nucleus, and cell adhesion during neuronal movement (*e.g.*, [52], [53], [54], [55]). These dynamic nuclear movements can lead to dramatic changes in cell shape: in many columnar epithelia including the developing Drosophila eye, the nucleus occupies the cell's region of greatest cross-sectional area and its movement can strongly distort the local environment. When harnessed in the fly pupal eye, this distortion provides necessary patterning information.

While the role of nuclear movements in cell fate specification and movements is only beginning to be appreciated, our results suggest that the resulting changes in cell packing—for example apical surface area—can strongly influence precise cell placement. We speculate that this mechanism is commonly employed in other emerging epithelia as well.

## Methods

### The Glazier-Graner-Hogeweg model

In the Glazier-Graner-Hogeweg (*GGH*) model used for our simulations [5], [18], [19], each cell exists as a group of points on an underlying lattice (for our simulation code and configuration files see Protocol S1). Exchanging lattice sites between adjacent cells at their boundaries randomly perturbs the shape and location of the cells. The probability that a proposed exchange occurs depends on its effect on the energy of the lattice, as determined by an energy function *H*. If a copy reduces the energy, we accept it with probability 1. Otherwise, we accept it with probability e^−Δ*H*/*T*^, where *T*, represents the intrinsic motility of the cells [5], [18], [19]. Each potential exchange is analyzed in a random order, with one exchange attempt for each lattice site defining the simulation's unit of time, a Monte Carlo Step (*MCS*) [5], [18], [19], here equivalent to about 2 seconds.

Each cell possesses a *cell type*, *τ*, which determines its physical properties and the contribution the cell makes to the overall energy of the lattice. Our simulations include energies due to cell adhesion and cell-area and cell-perimeter constraints.

Adhesion provides a mechanism for building complex structures, as well as for holding them together once they have formed. To represent variations in energy due to adhesion between cells of different types, we define a *boundary energy* that depends on 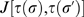, the *boundary energy per unit length* between two cells (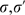) of given types (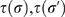) at a *link* (the interface between two neighboring lattice sites):
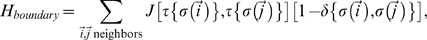
(1)where the sum is over all neighboring pairs of lattice sites 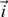 and 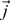 (the neighbor range may be greater than one lattice site), and the boundary-energy coefficients are symmetric,
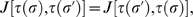
(2)
Table S3 lists the contact energies in our simulations.

To restrict variation of cell area we use an *area constraint*, of the following form:
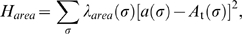
(3)where for cell 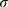, 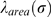 denotes the *inverse compressibility* of the cell, 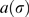 is the number of lattice sites in the cell (its *area*), and 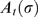 is the cell's *target area*. This constraint defines 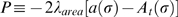 as the *pressure* inside the cell, that is, the susceptibility to grow or shrink in subsequent steps. A cell with 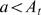 has a positive internal pressure and thus grows, while a cell with 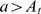 has a negative internal pressure and thus shrinks.

Since the experimental cells have nearly fixed amounts of cell membrane, we use a *surface-area constraint* of form:
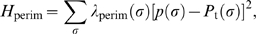
(4)where 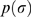 is the surface area of cell 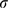, 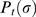 is its target perimeter. 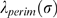 is its *inverse membrane compressibility*.

Adding the boundary energy and area constraint terms together (Equations (1), (3) and (4), we obtain the basic *GGH effective energy*:
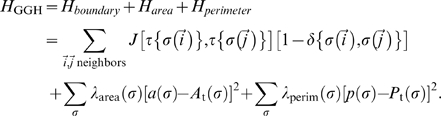
(5)


These equations are the 2D form of the canonical GGH equations [24]. The implementation of the GGH that we used for our simulations supports both 3D and 2D forms and detects which to use appropriately [24]. Table S5 lists the area and perimeter constraints used for our simulations. These constraints were selected to ensure that OCs maintained a roughly circular shape as observed *in vivo* [33] and allow minimal constraints on the shape of the IPCs.

We simulate cell death by setting 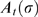 to 0 [39]. We used the open-source program, CompuCell3D, to implement the GGH (www.compucell3d.org).

### Implementation of apical surface expansion

CompuCell3D leaves implementation of dynamic properties of cells (*e.g.* cell growth rate, cell death rate, *etc.*) to the user. Our simulations implemented apical surface expansion as a CompuCell *steppable*, a callable software module executed a fixed number of times per MCS. In our implementation, we incremented the target area and/or target perimeter of cells of specific cell types by a defined amount until the target area reached a maximum. Table S1 lists the expansion parameters in our simulations.

### Creation of initialization files

Our goal was to determine the parameters necessary to obtain stable 3°s. *In vivo*, beginning at 23:00–24:00 hours APF, the IPCs of the eye reorganize so that each IPC touches at least two 1°s [11], [12], [16]. Since we were unsure if this event created a configuration necessary and sufficient for 3° formation or merely increased the number of 1°s contacted by each IPC, we created our initial simulation configuration by tracing micrographs of dissected pupal eyes staged between 23:00–24:30 APF and stained with antibodies recognizing an adherens-junction marker (Figure 2B and C). A custom program was used to convert tracings into a CompuCell3D-readable format. While we traced several eyes, we used a single representative tracing as the initialization file for each simulation in this study.

### Cell death

IPC cells die with a probability determined by their contact length (in lattice units) with neighboring OCs. If the contact area is greater than a *threshold length* (*L*), *P*(Death) = 0, otherwise:
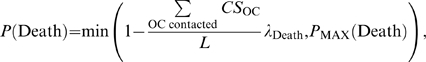
(6)where CS_OC_ denotes the contact length between an IPC cell and its OC neighbors, *λ*_Death_ scales this dependency and *P*_MAX_(Death) is the maximum probability an IPC cell will die per MCS. We evaluated each IPC cell for PCD once after each MCS and cells which died had their target area set to 0. All simulations used *λ*_Death_ = 1.2 and *P*_MAX_(Death) = 0.01.

### Cell measurements

We measured average OC and IPC sizes from images of live wild type retinae (*GMR-Gal4/UAS*-α*Catenin-GFP*) acquired as described in [33]. We outlined cells by hand and measured and recorded the area encompassing the cell using ImageJ (NIH). For each relevant case we measured at least 22 different OCs and 48 different IPCs.

### Scoring of simulations

To keep track of patterning in our simulations, we plotted the total number of cells, the number of 2° and 3° cells and the average area and perimeter for each cell type at 500 MCS intervals. We scored as 3°s all cells of type IPC that contacted exactly three OCs. We scored as 2°s all cells of type IPC that contacted exactly two OCs and two 3°s.

### Videos

We assembled videos from individual PNG files using the Mencoder program, part of the MPlayer software package (www.mplayerhq.hu) and converted them to MPEG format using ffmpeg (ffmpeg.mplayerhq.hu/).

### Generation and scoring of GMRGal4/UAS-DIAP1 eyes

To count the number of IPCs in the lattice surrounding a single ommatidium, we connected the centers of the surrounding six ommatidia to form an hexagonal outline on micrographs of dissected pupal eyes (Figure 5) staged to 41:00 hours APF at 25°C and stained with antibodies to DE-Cadherin (DSHB) as described in [27]. We counted cells within each hexagonal outline, arbitrarily scoring any cell that traversed the outline boundary as half a cell. To exclude any potential affects that bristle groups have on PCD [32] we analyzed only ommatidia with 3 correctly-positioned bristle groupings. We scored as 3°s all IPCs that contacted exactly three 1°s. We scored a total of 479 ommatidia and plotted the average number of 3°s per ommatidium.

### Generation of smurf over-expression clones

We heat shocked *hs-Flp; act>y>Gal4, UAS-GFP/+; UAS-smurf/+* pupae at 37°C for 20–30 min at 18:00–20:00 hr APF and dissected them at 40:00–42:00 hr APF. We used antibodies to Armadillo (N2 7A1, DSHB) to visualize adherens junctions as previously described [56].

### Transmission electron microscopy

We dissected wild type *Canton S* pupal eyes at 18:00 hr APF, incubated them in a glutaraldehyde/potassium permanganate fix and processed and imaged them as described previously [12].

## Supporting Information

Protocol S1 Simulation code and configuration files.
(0.96 MB ZIP)

Figure S1 Progressive differences between IPCs and OC surface proportions promoted 3° formation. Images captured at (A) 0 MCS and (A′) 50,000 MCS from a simulation with no expansion of half of the central OC, illustrated in red. The orange half of this OC was allowed to expand normally. The pattern fails to resolve correctly in IPCs surrounding this ommatidium (arrows in inset). Images captured at (B) 0 MCS and (B′) 50,000 MCS from a simulation with no expansion of the central OC, illustrated in red. The pattern fails to resolve correctly in IPCs surrounding this ommatidium (arrows in inset).
(6.44 MB TIF)

Table S1 Cell expansion parameters. Parameters used to expand the ommatidial cores over time. There is no constraint on IPC perimeter in order to allow IPCs to adopt polygonal shapes. In contrast, the OC is highly constrained to maintain a roughly circular shape. The target areas and perimeters are incremented as indicated and spaced by simulation time shown in the ΔMCS column.
(0.04 MB DOC)

Table S2 Parameters explored for model fitting. The number of potential parameter combinations was too large to explore exhaustively: we initially assumed a hierarchy of contact energies: *J*_OC, OC_>>*J*_OC, Medium_>>*J*_IPC, Medium_>*J*_IPC, IPC_≥*J*_IPC, OC_ and conducted sweeps of parameters outside the initial numbers to determine the range within which we observed patterning that mimicked a wild-type retina.
(0.05 MB DOC)

Table S3 Values for adhesions used in the simulations. Parameters used for the different adhesion configurations. Lower values indicate greater adhesion between cell types. OC:OC values were chosen to be large since OCs do not touch under wild-type conditions.
(0.04 MB DOC)

Table S4 Summary of simulation and in vivo results. Wild type is indicated by +++. Progressively more severe defects based on visual criteria are indicated by ++, +, −, −−, and −−−. ND indicates that the equivalent biological experiments have not been performed. Asterisks (**) indicate in vivo experiments reported in this paper.
(0.05 MB DOC)

Table S5 Initial cell area and perimeter constraints. Initial parameters used in simulations for cell perimeter and area of the two cell types. These are later modified over time, unless explicitly stated otherwise, as indicated in Table S1.
(0.04 MB DOC)

Video S1 Movie of a wild type simulation. This simulation was run over 50,000 MCS using optimized parameters. IPCs adhere to OCs with greater strength than to each other. The apical surface of the OCs expands and cell death begins at 10,000 MCS.
(5.86 MB MPG)

Video S2 Movie of a simulation of anti-preferential adhesion and reduced death (L = 10) run over 50,000 MCS. Cell death began at 10,000 MCS.
(5.97 MB MPG)

Video S3 Movie of a simulation with standard cell death conditions and expanding IPCs run over 50,000 MCS. In this simulation the IPCs increased in area along with the OCs. The tissue increases in size to the point that it wraps around the outer ‘medium’ due to the periodic boundary conditions in the simulation.
(6.38 MB MPG)
