# Supplementary material for: Computer Simulation of Cellular Patterning Within the Drosophila Pupal Eye
Source: PLoS Comput Biol. 2010 Jul 1;6(7):e1000841. doi: 10.1371/journal.pcbi.1000841 (PMC2895643; doi:10.1371/journal.pcbi.1000841)
Supplement: Protocol S1 — Simulation code and configuration files. (0.96 MB ZIP) [file pcbi.1000841.s001.zip › Installation of Drosophila Eye Specific CompuCell steppables.docx]

Installation of Drosophila Eye Specific CompuCell steppables

The eye simulations use several CompuCell3D steppables. These are all written in C++ rather than Python and thus must be compiled. They are located within the folder called CompuCellAdditions. These were used with CompuCell3D 3.0.15 and earlier. Compatibility with later versions is untested.

Proceed as follows for installation of this code.

1. Download CompuCell and unpack the zipped file
2. Copy the MSInterface folder into the CompuCell3D*/CompuCell3D/ folder.
3. Copy the contents of the steppables folder into the CompuCell3D*/CompuCell3D/steppables/ folder
4. Append the following lines to the CompuCell3D*/CompuCell3D/steppables/CMakeLists.txt file:

ADD_SUBDIRECTORY(CellGrowth)

ADD_SUBDIRECTORY(CellKiller)

ADD_SUBDIRECTORY(CellTypeDataOutput)

ADD_SUBDIRECTORY(ContactAbsentKiller)

ADD_SUBDIRECTORY(EdgeSavior)

ADD_SUBDIRECTORY(LocalFlexInitializer)

ADD_SUBDIRECTORY(MasterSteppable)

ADD_SUBDIRECTORY(SurfaceAreaKiller)

1. Continue by following the CompuCell installation directions.

Manifest

| Filename | Description |
| --- | --- |
| 24_25apf_single_nonexpanding.pif | Initial configuration for asymmetric expansion simulations |
| 24_2apf795cells.pif | Initial configuration for all wild-type based simulations |
| 24rst.pif | Initial configuration for roughest simulation |
| AntiBaseline.xml | Wild-type simulation, but with Anti-preferential adhesion |
| AntiDeath=10.xml | Weakened Death (L=10) with Anti-preferential adhesion |
| FlatBaseline.xml | Wild-type simulation, but with flat adhesion between OC and IPCs |
| FlatDeath=10.xml | Weakened Death (L=10) with flat adhesion between OC and IPCs |
| NoDeath.xml | Flat adhesion but with no cell death |
| NoDeathNoGrowth.xml | Flat adhesion between OC and IPCs with no cell death or OC expansion |
| Nonexpanding.xml | Single half of OC does not expand |
| PrefAdhNogrowthnodeath.xml | Wild-type but with no death |
| PrefAdhnodeath.xml | Wild-type |
| PrefBaseline.xml | Wild-type but with reduced death (L=10_ |
| Rst.xml | Simulation of Rst mutant tracing with wild-type parameters |
| Rst_bigIPC.xml | Simulation of Rst mutant tracing with expanding IPCs |
| growIPCs.xml | Wild-type with expanding IPCs |
| weakDeathSeries0.xml | Flat adhesion with weak death L=2 |
| weakDeathSeries1.xml | Flat adhesion with weak death L=4 |
| weakDeathSeries2.xml | Flat adhesion with weak death L=6 |
| weakDeathSeries3.xml | Flat adhesion with weak death L=8 |
| weakDeathSeries4.xml | Flat adhesion with weak death L=10 |
| weakDeathSeries5.xml | Flat adhesion with weak death L=12 |
| weakDeathSeries6.xml | Flat adhesion with weak death L=14 |
| weakDeathSeries7.xml | Flat adhesion with weak death L=16 |
| weakDeathSeries8.xml | Flat adhesion with weak death L=18 |
