# Supplementary figures and images for: Computer Simulation of Cellular Patterning Within the Drosophila Pupal Eye
Source: PLoS Comput Biol. 2010 Jul 1;6(7):e1000841. doi: 10.1371/journal.pcbi.1000841 (PMC2895643; doi:10.1371/journal.pcbi.1000841)

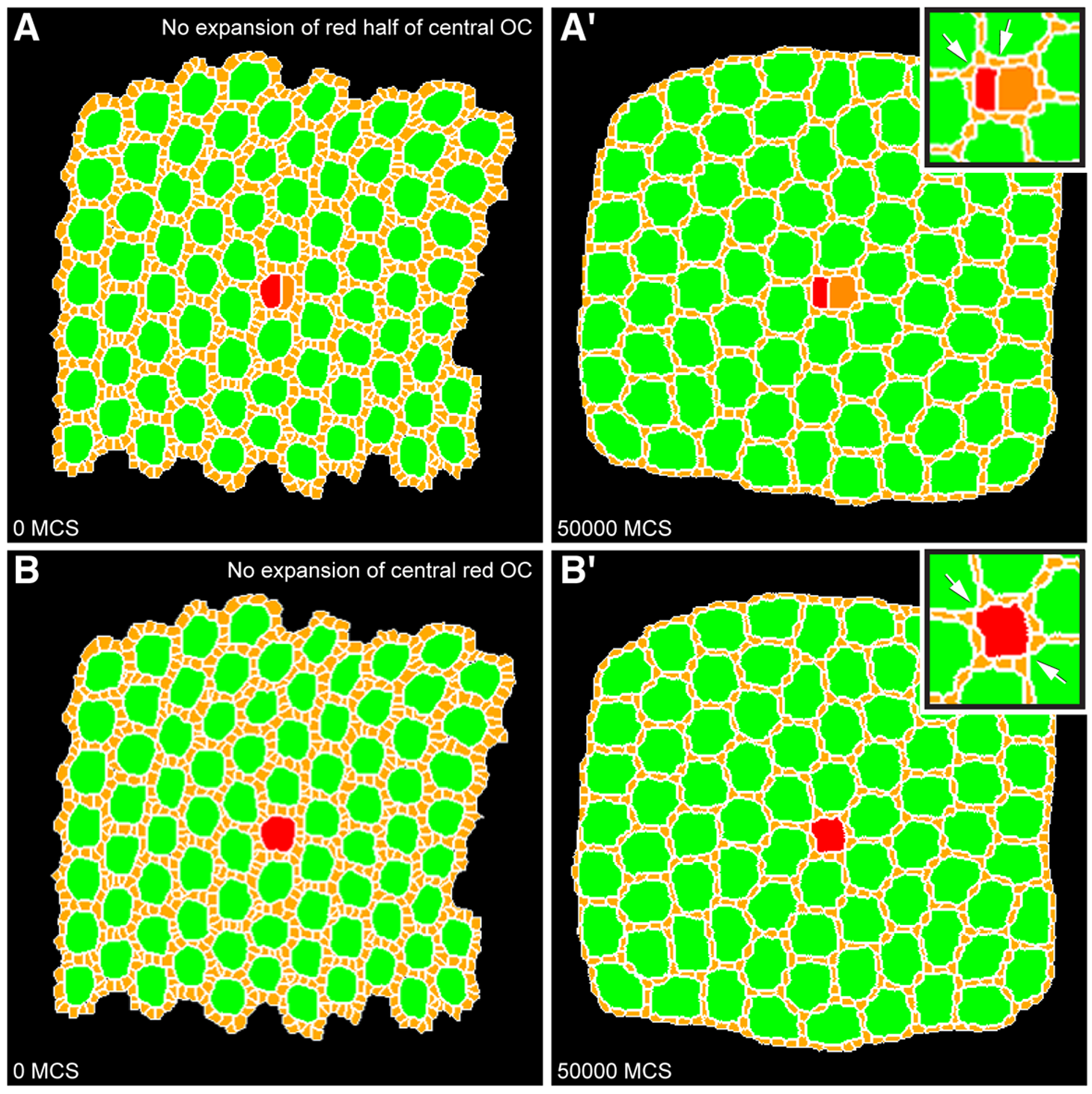

Supplement: Figure S1 — Progressive differences between IPCs and OC surface proportions promoted 3° formation. Images captured at (A) 0 MCS and (A′) 50,000 MCS from a simulation with no expansion of half of the central OC, illustrated in red. The orange half of this OC was allowed to expand normally. The pattern fails to resolve correctly in IPCs surrounding this ommatidium (arrows in inset). Images captured at (B) 0 MCS and (B′) 50,000 MCS from a simulation with no expansion of the central OC, illustrated in red. The pattern fails to resolve correctly in IPCs surrounding this ommatidium (arrows in inset). (6.44 MB TIF) [file pcbi.1000841.s002.tif]
