# Supplementary material for: Computer Simulation of Cellular Patterning Within the Drosophila Pupal Eye
Source: PLoS Comput Biol. 2010 Jul 1;6(7):e1000841. doi: 10.1371/journal.pcbi.1000841 (PMC2895643; doi:10.1371/journal.pcbi.1000841)
Supplement: Table S1 — Cell expansion parameters. Parameters used to expand the ommatidial cores over time. There is no constraint on IPC perimeter in order to allow IPCs to adopt polygonal shapes. In contrast, the OC is highly constrained to maintain a roughly circular shape. The target areas and perimeters are incremented as indicated and spaced by simulation time shown in the ΔMCS column. (0.04 MB DOC) [file pcbi.1000841.s003.doc]

**Supplemental Table 1**

| **Table S1. Cell Expansion Parameters** | | | | | |
| --- | --- | --- | --- | --- | --- |
| Cell Type | Final $A_{t}\left( s \right)$ | Δ_A_ | Final $P_{t}\left( s \right)$ | Δ_P_ | ΔMCS |
| OC | 1000 | 1 | 150 | 1 | 125 |
| IPC | 120 | 1 | None | None | 800 |
