# Supplementary material for: Computer Simulation of Cellular Patterning Within the Drosophila Pupal Eye
Source: PLoS Comput Biol. 2010 Jul 1;6(7):e1000841. doi: 10.1371/journal.pcbi.1000841 (PMC2895643; doi:10.1371/journal.pcbi.1000841)
Supplement: Table S2 — Parameters explored for model fitting. The number of potential parameter combinations was too large to explore exhaustively: we initially assumed a hierarchy of contact energies: J OC, OC>>J OC, Medium>>J IPC, Medium>J IPC, IPC≥J IPC, OC and conducted sweeps of parameters outside the initial numbers to determine the range within which we observed patterning that mimicked a wild-type retina. (0.05 MB DOC) [file pcbi.1000841.s004.doc]

**Supplemental Table 2**

| **Table S2. Parameters Explored for Model Fitting** | | | | | | | |  |
| --- | --- | --- | --- | --- | --- | --- | --- | --- |
| Results | T | J_Medium, Medium_ | J_OC,Medium_ | J_IPC,Medium_ | J_OC,OC_ | J_OC,IPC_ | J_IPC,IPC_ | |
| ++ | 25 | 0 | 120 | 60 | 1200 | 35 | 55 | |
| + | 60 | 0 | 120 | 60 | 1200 | 5-20(Δ5) | 5-20(Δ5) | |
| +++ | 60 | 0 | 120 | 60 | 1200 | 25-40(Δ5) | 25-40(Δ5) | |
| +++ | 60 | 0 | 120 | 60-120  (Δ30) | 1200 | 40-100(Δ30) | 40-100(Δ30) | |
| ++ | 60 | 0 | 120 | 150 | 1200 | 130 | 130 | |
| + | 60 | 0 | 120 | 180-210  (Δ30) | 1200 | 160-190(Δ30) | 160-190(Δ30) | |
| +++ | 70-100  (Δ10) | 0 | 120 | 60 | 1200 | 35 | 55 | |
| ++ | 100-170  (Δ10) | 0 | 120 | 60 | 1200 | 35 | 55 | |
| +++ | 70 | 0 | 70-120  (Δ10) | 60 | 70-570  (Δ100) | 35 | 55 | |
