# Supplementary material for: Computer Simulation of Cellular Patterning Within the Drosophila Pupal Eye
Source: PLoS Comput Biol. 2010 Jul 1;6(7):e1000841. doi: 10.1371/journal.pcbi.1000841 (PMC2895643; doi:10.1371/journal.pcbi.1000841)
Supplement: Table S3 — Values for adhesions used in the simulations. Parameters used for the different adhesion configurations. Lower values indicate greater adhesion between cell types. OC:OC values were chosen to be large since OCs do not touch under wild-type conditions. (0.04 MB DOC) [file pcbi.1000841.s005.doc]

**Supplemental Table 3**

| **Table S3. Values for Adhesions Used in the Simulations** | | | | | | |
| --- | --- | --- | --- | --- | --- | --- |
| Type of Adhesion | J_Medium, Medium_ | J_OC,Medium_ | J_IPC,Medium_ | J_OC,OC_ | J_OC,IPC_ | J_IPC,IPC_ |
| Preferential (wild type) | 0 | 120 | 60 | 1200 | 35 | 55 |
| Flat | 0 | 120 | 60 | 1200 | 40 | 40 |
| Anti-Preferential | 0 | 120 | 60 | 1200 | 55 | 35 |
