# Supplementary material for: Computer Simulation of Cellular Patterning Within the Drosophila Pupal Eye
Source: PLoS Comput Biol. 2010 Jul 1;6(7):e1000841. doi: 10.1371/journal.pcbi.1000841 (PMC2895643; doi:10.1371/journal.pcbi.1000841)
Supplement: Table S4 — Summary of simulation and in vivo results. Wild type is indicated by +++. Progressively more severe defects based on visual criteria are indicated by ++, +, −, −−, and −−−. ND indicates that the equivalent biological experiments have not been performed. Asterisks (**) indicate in vivo experiments reported in this paper. (0.05 MB DOC) [file pcbi.1000841.s006.doc]

| **Supplemental Table S4**  **Table S4. Summary of simulation and *in vivo* results** | | | | | | | | | |
| --- | --- | --- | --- | --- | --- | --- | --- | --- | --- |
| **Simulation Results** | | | | | **Biological Behaviors** | | | | |
| **genotype** | **2°s** | **3°s** | **cell death** | **overall hexagonal patterning** | **genotype** | **2°s** | **3°s** | **cell death** | **overall patterning** |
| wild type | **+++** | **+++** | **+++** | **+++** | wild type | **+++** | **+++** | **+++** | **+++** |
| altered cell motility (*T*) | **+++** | **+++** | **+++** | **+++** | altered cell motility (*T*) | ND | ND | ND | ND |
| decreased cell death | **--** | **--** | **---** | **+** | decreased cell death | **--** | **--** | **---** | **+** |
| preferential adhesion | **+++** | **+++** | **+++** | **+++** | preferential adhesion (wild type) | **+++** | **+++** | **+++** | **+++** |
| flat adhesion | **++** | **++** | **++** | **++** | *rst*  *hs-rst* | **---** | **---** | **---** | **---** |
| anti-preferential adhesion | **++** | **++** | **++** | **++** | anti-preferential adhesion | ND | ND | ND | ND |
| asymmetric cell expansion | **---** | **---** | **---** | **---** | asymmetric cell expansion** | **---** | **---** | **---** | **---** |
| symmetric cell expansion | **-** | **-** | **-** | **-** | symmetric cell expansion** | **-** | **-** | **-** | **-** |
