# Supplementary material for: Computer Simulation of Cellular Patterning Within the Drosophila Pupal Eye
Source: PLoS Comput Biol. 2010 Jul 1;6(7):e1000841. doi: 10.1371/journal.pcbi.1000841 (PMC2895643; doi:10.1371/journal.pcbi.1000841)
Supplement: Table S5 — Initial cell area and perimeter constraints. Initial parameters used in simulations for cell perimeter and area of the two cell types. These are later modified over time, unless explicitly stated otherwise, as indicated in Table S1. (0.04 MB DOC) [file pcbi.1000841.s007.doc]

**Supplemental Table S5**

| **Table S5. Initial Cell Area and Perimeter Constraints** | | | | |
| --- | --- | --- | --- | --- |
| Cell Type |  |  |  |  |
| OC | 550 | 50 | 120 | 5 |
| IPC | 60 | 2 | None | None |
